# Enhancement of Microwave Heating Technology for Emulsified Asphalt Mixtures Using SiC-Fe_3_O_4_ Composite Material

**DOI:** 10.3390/ma17184572

**Published:** 2024-09-18

**Authors:** Sheng Xu, Wen Xu, Yixing Chen, Jiaqi Li, Yueguang Li

**Affiliations:** School of Transportation and Logistics Engineering, Wuhan University of Technology, Wuhan 430063, China

**Keywords:** emulsified asphalt mixture, SiC-Fe_3_O_4_ composite material, microwave absorption performance, microwave curing performance

## Abstract

The application of microwave heating technology can significantly enhance the water evaporation rate of emulsified asphalt mixtures post paving. To improve the microwave absorption and curing performance of these mixtures, SiC-Fe_3_O_4_ composite material (SF) was incorporated. This addition aims to enhance the microwave absorption efficiency and accelerate the curing process of emulsified asphalt mixtures under microwave heating. This study begins with an analysis of the microwave absorption principles pertinent to emulsified asphalt mixtures. Subsequently, the microwave heating temperature fields of ordinary emulsified asphalt mixture (EAM), SiC emulsified asphalt mixture (S-EAM), Fe_3_O_4_ emulsified asphalt mixture (F-EAM), and SiC-Fe_3_O_4_ emulsified asphalt mixture (SF-EAM) were simulated using COMSOL Multiphysics finite element software (COMSOL 6.2). The early strength variations in SF-EAM under different microwave heating durations were then examined through adhesion tests, leading to the proposal of a microwave heat curing process for SF-EAM. Finally, the wear resistance, water damage resistance, rutting resistance, and skid resistance of SF-EAM post-microwave curing were evaluated through wet wheel wear tests, wheel track deformation tests, and road friction coefficient tests. The results indicate that the optimal microwave heating time is 90 s, with the microwave absorption performance of the materials ranked as follows: EAM, S-EAM, F-EAM, and SF-EAM, from lowest to highest. The road performance of SF-EAM complies with specification requirements, and its wear resistance, water damage resistance, and rutting resistance are notably improved after microwave heating.

## 1. Introduction

With the continuous improvement of the national highway network, various levels and types of highways have been constructed and opened to traffic, leading to a saturation in China’s highway construction. Consequently, the focus has shifted from construction to maintenance. Among various road maintenance methods, emulsified asphalt mixture (EAM) plays a crucial role. It is commonly used in maintenance techniques such as slurry sealing, micro-surfacing, the cold patching of road surfaces, and asphalt cold recycling technology. The development of road maintenance and construction technology based on emulsified asphalt mixture can save energy, and at the same time, the construction technology without heating raw materials can reduce the emission of toxic gasses, which is in line with the strategic requirements of energy conservation and environmental protection in today’s world [1]. However, a contradiction exists between “construction slow cracking” and “molding fast setting” in the application of emulsified asphalt. During construction, emulsified asphalt and mineral aggregate require time for thorough mixing, and the mixture needs time to be spread on the pavement. During this process, the mixture must maintain fluidity [2]. Once spread, it is necessary to quickly evaporate the moisture and solidify the mixture to enable quick traffic opening [3]. Therefore, in-depth research is required to address the challenges of rapid solidification and the formation of emulsified asphalt mixtures post mixing and paving.

The solidification process of emulsified asphalt mixtures involves breaking the emulsion, solidifying the mixture, and achieving a certain strength. Klaila et al. first proposed the concept of microwave demulsification in 1984, successfully conducting feasibility experiments using microwaves to dehydrate crude oil [4]. Later, Fang et al. suggested that microwave radiation could break oil–water solid emulsions, achieving faster separation than gravity settling and conventional heating methods [5]. Abdurrahman et al. found that microwave radiation is a dielectric heating technique characterized by penetration, rapid, uniform, and selective heating, making it suitable for emulsion-breaking processes [6]. Thus, applying microwave heating technology to demulsify emulsified asphalt and solidify the mixture is feasible.

The inherent microwave absorption performance of the original materials in emulsified asphalt mixtures is relatively poor. Enhancing the mixture’s microwave absorption by adding microwave absorbing materials can increase the microwave curing speed. Microwave absorbing materials can be categorized based on their loss mechanisms into electrical loss type and magnetic loss type [7] materials.

Guan et al. noted that Fe_3_O_4_ in natural magnetite powder exhibits good electromagnetic properties. Replacing mineral powder with magnetite in asphalt mixtures can improve microwave absorption [8]. Chen et al. studied the microwave absorption properties of SiC and boron-doped SiC, finding that boron-doped SiC shows superior microwave absorption performance in the 2–18 GHz frequency range. At 14 GHz and a material thickness of 1.5 mm, the maximum microwave reflection loss is −37.94 dB [9]. Liu et al. proposed that a composite material comprising SiC (an electrical loss-type material) and Fe_3_O_4_ (a magnetic loss-type material) can significantly enhance the microwave absorption performance of asphalt mixtures [10]. Consequently, SiC can be used as a standalone microwave absorbing material or combined with other materials for improved performance.

Additionally, incorporating SiC into asphalt mixtures can improve their road performance. Sun et al. mentioned that nano SiC has excellent thermal conductivity and oxidation resistance at high temperatures. Experiments showed that adding 100 nm nano SiC at 6% content maximizes asphalt aging resistance and improves water damage resistance [11]. Wang et al. compared the early road performance of cold recycled emulsified asphalt mixtures treated at room temperature, in an oven, and microwaved. They used environmental scanning electron microscopy and optical microscopy to study the morphology and emulsion-breaking behavior. Gray relational analysis was employed to compare the influence of microwave radiation time on the early road performance of cold recycled emulsified asphalt mixtures [12].

Dai et al. analyzed the early strength and road performance of cold recycled emulsified asphalt mixtures heated by microwave, finding microwave heating effective in enhancing early strength. Based on experimental results, a heating power of 800 W and a microwave heating time of 15 min were recommended. The study demonstrated that microwave heat curing rapidly increases early strength, making it superior to room temperature and oven curing [13].

Wang et al. studied the influence of the microwave heating time, power, water–cement ratio, and asphalt–cement ratio on the early strength of cement–asphalt lotion mixtures. They concluded that the total microwave energy is composed of power and radiation time, and higher total microwave energy results in higher surface temperature [14]. However, excessively long microwave radiation times can reduce mixture strength, necessitating a balanced approach to improve early strength.

Although prior research has primarily focused on strength and road performance concerning microwave emulsion breaking and curing, few studies have emphasized incorporating microwave absorbing materials to enhance absorption performance. Few have explored enhancing microwave absorption performance through both electrical and magnetic losses simultaneously. This study employed theoretical research, numerical simulation, and laboratory experiments to investigate the microwave absorption and curing performance of SiC-Fe_3_O_4_ emulsified asphalt mixtures, aiming to apply improved microwave heating technology in practical engineering.

**Figure 1:**
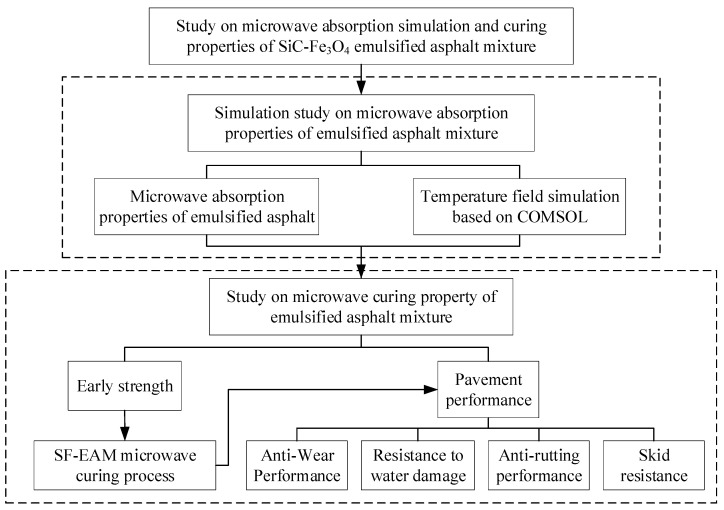
Technical roadmap.

The aim of this study was to improve the microwave absorption and curing performance of emulsified asphalt mixtures. This research started by analyzing the microwave absorption performance of each component material in emulsified asphalt mixtures, leading to the introduction of the microwave absorbing material SF. Subsequently, COMSOL Multiphysics finite element software was used to simulate the microwave heating temperature field of different emulsified asphalt mixtures, evaluating the microwave absorption performance through temperature field changes. Early strength variations in SF-EAM under different microwave heating times were studied through adhesion tests to determine a reasonable microwave heating time and propose a curing method. Finally, the wear resistance, water damage resistance, rutting resistance, and skid resistance of SF-EAM cured by microwave heating were evaluated using wet wheel wear tests, wheel track deformation tests, and road friction coefficient tests. The technical roadmap is shown in Figure 1.

## 2. Materials and Methods

### 2.1. Materials

#### 2.1.1. Modified Emulsified Asphalt

The emulsified asphalt mixture prepared in this study had to meet the performance requirements for micro-surfacing according to the technical requirements outlined in the “Guidelines for Micro Surfacing and Slurry Seal Technology” [15] (hereinafter referred to as the “Guidelines”) and the test method referred to the “Standard Test Methods of Bitumen and Bituminous Mixtures for Highway Engineering” [16]. The main technical indicators are presented in Table 1.

#### 2.1.2. Mineral Materials

The coarse and fine aggregates used in this study were ordinary basalt aggregates and the test method referred to the “Test Methods of Aggregates for Highway Engineering” [17]. The main technical indicators are shown in Table 2.

#### 2.1.3. Filler

The first type of filler used in this study was ordinary mineral powder. The test methods used are referred to as the “Test Methods of Aggregates for Highway Engineering” and “Test Methods of Soils for Highway Engineering” [17,18]. According to the technical requirements for mineral powder outlined in the “Technical Specification for Construction of Highway Asphalt Pavement” [19], the main technical indicators are presented in Table 3.

The second type of filler used in this study was ordinary Portland cement. The test method used is referred to as the “Testing Methods of Cement and Concrete for Highway Engineering” [20] According to the technical requirements for cement specified in the “Technical Rules for Construction of Highway Cement Concrete Pavement” [21], the main technical indicators are presented in Table 4.

#### 2.1.4. Additive Materials

The SiC used in this study was high-purity silicon carbide powder produced by Nan gong Yingtai Metal Materials Co., Ltd. (Xingtai, China). The SiC content in the silicon carbide powder exceeded 99%. The primary function of SiC is to enhance the microwave absorption performance of the emulsified asphalt mixture by improving its electrical loss characteristics. The test method used is referred to as the “Test Methods of Aggregates for Highway Engineering” and “Test Methods of Soils for Highway Engineering” [17,18]. The basic technical performance of SiC was evaluated according to the technical requirements for mineral powder, and the test results are shown in Table 5.

The Fe_3_O_4_ used in this study was high-purity ferric oxide powder produced by Hebei Keze Metal Materials Co., Ltd. (Xingtai, China). The Fe_3_O_4_ content in the ferric oxide powder exceeded 99%. The primary function of Fe_3_O_4_ is to enhance the microwave absorption performance of the emulsified asphalt mixture by improving its magnetic loss characteristics. The basic technical performance of Fe_3_O_4_ was evaluated according to the technical requirements for mineral materials, and the test results are shown in Table 6.

Based on the characteristics of two materials with similar densities and no chemical reactions with each other [22,23], the method for preparing SiC-Fe_3_O_4_ composite materials from SiC and Fe_3_O_4_ in this study involved mixing a specified mass of SiC powder and Fe_3_O_4_ powder in a stirring cup, stirring with a glass rod until the two materials were completely and evenly mixed, and then placing them into a sealed bag for later use.

#### 2.1.5. Grading

**Figure 2:**
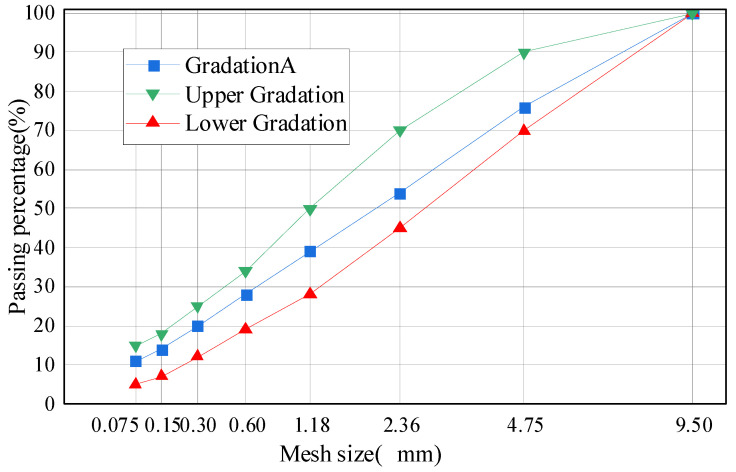
Grading curve.

This study used the commonly applied MS-3 gradation for micro-surfacing, with the range shown in Figure 2. Through moderately adjusting the pass rate of the mixture through each particle size sieve, the gradation curve of the designed mixture was made to closely align with the gradation curve obtained using the Taylor formula when *n* = 0.4. Ultimately, gradation A of this study was designed [24]. The scope is shown in Figure 2.

### 2.2. Experimental Equipment and Methods

#### 2.2.1. Microwave Heating Equipment and Principle

**Figure 3:**
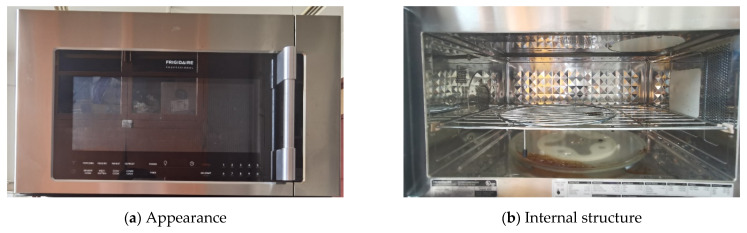
(**a**) Appearance and (**b**) interior of microwave oven (model FPBM3077RFA).

**Figure 4:**
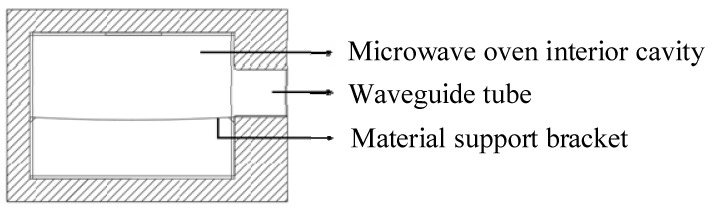
Internal structure diagram of microwave oven.

The microwave heating equipment used in this study was a large multifunctional microwave oven, model FPBM3077RFA. The appearance and interior are shown in Figure 3, and the internal structure diagram is shown in Figure 4. The waveguide is located on the right side of the microwave oven, which is suitable for the microwave heating of common emulsified asphalt mixture specimens. The maximum heating power of the microwave is 1000 W, with a frequency of 2.45 GHz, and the minimum accuracy of the microwave heating time is 1 s. The standard curing box is a constant temperature and humidity box, model LHS-100CL. The curing temperature range of the standard curing box is 0–85 °C, and the curing relative humidity range is 45–98%.

Changes in current or charge within a charged system can induce variations in the electromagnetic field. A changing electric field generates a changing magnetic field, and vice versa. These alternating electric and magnetic fields continuously propagate in the form of waves, known as electromagnetic waves. Each type of electromagnetic wave possesses a specific frequency, defined as the number of periodic changes in the electromagnetic field per unit time. Microwaves are a type of electromagnetic wave with a frequency range of 300 MHz to 300 GHz and a wavelength range of 1 mm to 1 m, categorized into three bands: decimeter wave, centimeter wave, and millimeter wave [25].

When microwaves encounter different materials during transmission, various phenomena occur, primarily reflection, penetration, and absorption. These phenomena are influenced by factors such as the dielectric constant and electromagnetic loss coefficient of the materials. Based on the microwave reflection, transmission, and absorption characteristics of different materials, commonly used materials are categorized into conductors, insulators, dielectrics, and magnetic compounds.

Under the influence of electromagnetic fields generated by microwaves, media can convert electromagnetic energy into thermal energy. The efficiency of media in absorbing microwaves is related to their complex permittivity and complex permeability, generally represented by Equations (1) and (2) [26].

(1) $$\varepsilon =\varepsilon^{\prime }-j\varepsilon^{\prime \prime }=\varepsilon_{0}\varepsilon_{r}=\varepsilon_{0}(\varepsilon_{r}^{\prime }-\varepsilon_{r}^{\prime \prime })$$

where ε is the dielectric constant of the material, F/m; $\varepsilon_{0}$ is the permittivity of vacuum, with a value of 8.854 × 10^−128.854^; $\varepsilon_{r}$ is the relative permittivity of the material; $\varepsilon_{r}^{\prime }$ is the real part of the relative permittivity; $\varepsilon_{r}^{\prime \prime }$ is the imaginary part of the relative permittivity; and j is the imaginary unit.

(2) $$\mu =\mu^{\prime }-j\mu^{\prime \prime }=\mu_{0}\mu_{r}=\mu_{0}(\mu_{r}^{\prime }-j\mu_{r}^{\prime \prime })$$

where μ is the complex permeability of the material, H/m; $\mu_{0}$ is the permeability of vacuum, with a value of 4π × 10^−7^ H/m; $\mu_{r}$ is the relative permeability of the material; $\mu_{r}^{\prime }$ is the real part of the relative permeability; and $\mu_{r}^{\prime \prime }$ is the imaginary part of the relative permeability. The power absorbed by the material from the microwave is given by Equations (3) and (4) [27].

(3) $$P=0.556\;fE^{2}\varepsilon_{r}\tan\delta \times 10^{-12}$$

where P is the power absorbed per unit volume of the material, W/cm^3^; f is the operating frequency of the microwave, Hz; E is the magnitude of the electric field, W/cm; and δ is the loss angle of the material.

(4) $$\tan\delta =\tan\delta_{E}+\tan\delta_{M}=\frac{\varepsilon^{\prime \prime }}{\varepsilon^{\prime }}+\frac{\mu^{\prime \prime }}{\mu^{\prime }}$$

where $\delta_{E}$ is the electric loss angle of the material, and $\delta_{M}$ is the magnetic loss angle of the material.

According to Equations (3) and (4), it can be seen that the absorption power of materials for microwaves is influenced by two factors: internal and external. Among them, the working frequency f and electric field strength E of the microwave are external factors that depend on the properties of the microwave field. The higher the working frequency and electric field strength, the greater the absorption power of the microwave. The relative dielectric constant ($\varepsilon_{r}$) and loss angle (δ) of the material are internal factors that depend on the properties of the material. Specifically, $\varepsilon^{\prime \prime }$ and $\mu^{\prime }$ are the real parts of the dielectric constant and magnetic permeability, respectively, characterizing the energy storage capacity of the material, such as electrical energy and magnetic energy. The imaginary parts $\varepsilon^{\prime \prime }$ and $\mu^{\prime \prime }$ represent the material’s loss factor, reflecting the dielectric material’s ability to dissipate electromagnetic waves.

A good material for microwave absorption not only needs to exhibit strong microwave loss capabilities but also needs to allow microwaves to penetrate the material as much as possible, minimizing reflection at the material interface. The reflection loss is given by Equations (5) and (6). The smaller the reflection loss and microwave reflectivity when microwaves enter the material, the better the microwave absorption performance of the material [28].

(5) $$R_{L}=20\mathrm{lg}\left|\frac{Z_{in}-Z_{0}}{Z_{in}+Z_{0}}\right|$$

where $R_{L}$ is the reflection loss, in dB; $Z_{in}$ is the impedance of the microwave in free space, in ohms (Ω); and $Z_{0}$ is the impedance at the interface between free space and the material, in ohms (Ω).

(6) $$Z_{in}=Z_{0}\sqrt{\frac{\mu }{\varepsilon }}\tanh\left[j(2\pi ft/c)\sqrt{\mu \varepsilon }\right]$$

where c is the speed of light, in m/s; f is the operating frequency of the microwave, in Hz; and t is the thickness of the material, in meters (m).

According to Equation (3), to increase microwave absorption power, the electric field strength E or microwave frequency (f) can be increased, and the relative dielectric constant (*ε_r_*) or loss angle (*δ*) of the material can also be improved by changing its properties. However, the electric field strength cannot be increased indefinitely, as high electric field strength can lead to electrode breakdown [25], which is not conducive to heating materials. In addition, blindly increasing the frequency is also ineffective because when electromagnetic waves penetrate into the medium, some of the energy will be consumed and converted into heat energy, causing the electric field strength to decay according to a certain law.

The distance from the surface at which the microwave energy decreases to its original maximum value is defined as the penetration depth. When the dielectric loss factor (D) of the material is small, the penetration depth (D) can be expressed by Equation (7) [29].

(7) $$D=\frac{\lambda \sqrt{\varepsilon^{\prime }}}{2\pi \varepsilon^{\prime \prime }}$$

where D is the penetration depth of the microwave, in cm, and λ is the wavelength of the microwave, in cm.

According to Equation (7), the microwave penetration depth (D) and microwave wavelength (λ) are of the same order of magnitude. The wavelength range of microwaves is usually 1 mm to 1 m, allowing most materials to be uniformly heated by microwaves. When using higher frequency electromagnetic waves to heat materials, the wavelength of the electromagnetic waves becomes shorter. Although high-frequency electromagnetic fields can increase the absorption power of the medium, the short wavelength and small penetration depth of the waves prevent comprehensive and uniform heating.

Therefore, altering the composition of the mixture and improving the electromagnetic parameter properties of the material are effective ways to enhance the microwave absorption power of the material.

#### 2.2.2. Cohesion Test

**Figure 5:**
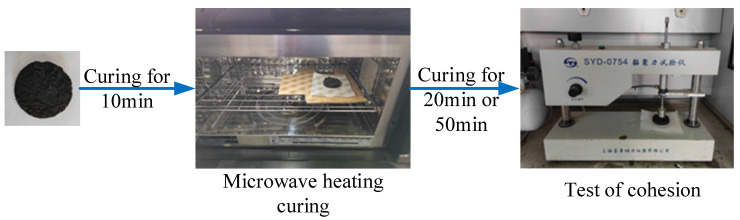
Adhesion test after microwave heat curing.

This section evaluates the early strength of emulsified asphalt mixtures through cohesion tests. Under the same conditions, the adhesive strength and molding state of SF-EAM specimens cured by heating with different microwave times may vary. When the microwave heating time is insufficient, the strength of the emulsified asphalt mixture cannot meet the standard specifications. Conversely, when the microwave heating time is too long, the asphalt in the emulsified asphalt mixture will overheat and age, affecting the strength. Therefore, by designing the adhesion test of SF-EAM, its reasonable microwave heating time can be determined. After the SF-EAM adhesive strength test specimen is formed, it needs to be microwave-heated and cured before testing the adhesive strength. Therefore, this section includes some adjustments and modifications to the cohesion test steps specified in the standards. The specific test steps are as follows:

1. Prepare a specific quantity of mineral materials, modified emulsified asphalt, cement, water, SiC, Fe_3_O_4_, and other materials. Prepare SiC-Fe_3_O_4_ emulsified asphalt mixture (SF-EAM) specimens and ordinary emulsified asphalt mixture (EAM) specimens without composite materials. Set EAM as the control group. The SF content in SF-EAM is 4%, and the composite ratio of the two materials is SiC = 1:1.
2. Prepare standard specimens for adhesive strength testing according to the specifications. Divide the demolded specimens into 6 groups and place them in a laboratory environment at room temperature for 10 min. Then, place them in a microwave oven with a power of 1000 W for 0 s, 30 s, 60 s, 90 s, 120 s, and 150 s. After heating, remove the specimens and place them at room temperature for 20 min (for 30 min adhesive strength testing) or 50 min (for 60 min adhesive strength testing). The curing methods for each group of specimens are shown in Table 7.

3. Place the cured specimen on the testing platform of the cohesion tester and measure the cohesion according to the specifications, with an accuracy of 0.1 N·m.
4. Repeat steps (1) to (3) to test the adhesion of EAM and SF-EAM under different microwave exposure times, as shown in Figure 5.

#### 2.2.3. Microwave Heat Curing Process of SF-EAM

This section proposes the microwave heat curing process for SF-EAM, which uses microwave heating technology to heat and cure emulsified asphalt mixture specimens, thereby improving the early strength of newly mixed and formed specimens. The specific steps are as follows:

1. Prepare raw materials according to the ratio of mineral aggregate–emulsified asphalt–water–cement = 100:12.8:6:1 [24]. The mineral aggregate consists of coarse aggregate, fine aggregate, and mineral powder, with coarse and fine aggregates accounting for 89% and mineral powder accounting for 11%. The grading of each component is shown in Figure 2.
2. Mix SiC and Fe_3_O_4_ evenly in a 1:1 ratio to produce SF, and replace 4% of the mineral powder by mass with SF.
3. First, mix the aggregate, mineral powder, SF, and cement at room temperature. Then, add water to the mixture, and finally, add emulsified asphalt and stir evenly. The total mixing time should not exceed 60 s.
4. Pour the mixed emulsified asphalt mixture into the test mold, lightly compact, and scrape it flat.
5. Demold the specimen, and then place it in the laboratory at room temperature for 10 min for curing. Finally, place it in a microwave oven and heat it with a microwave power of 600–1000 W to cure. During heating, adjust the microwave power according to different specimens to ensure that the maximum surface temperature of the specimen does not exceed 150 °C.
6. Remove the heated specimen and continue with subsequent tests according to the standard specifications.

#### 2.2.4. Wet Wheel Wear Test

This section evaluates the wear resistance and water damage resistance of SF-EAM through wet wheel wear tests after 1 h and 6 days of water immersion. Wear and tear refer to the phenomenon where the road surface is gradually damaged due to repeated friction caused by loads during use. The maintenance pavement made of emulsified asphalt mixture as a raw material usually has high wear resistance, which is generally determined by the material properties and maintenance methods of the pavement. Firstly, SF-EAM contains two types of doping materials, SiC and Fe_3_O_4_, which can affect the gradation of the mixture and thus affect the wear resistance of SF-EAM. Secondly, the specimens cured by microwave heating have stronger adhesion between materials, which also affects the wear resistance of SF-EAM. Two sets of control tests were established:

1. The first set includes EAM and SF-EAM specimens subjected to indoor curing, with EAM specimens as the control group.
2. The second set includes SF-EAM specimens subjected to both indoor curing and microwave heat curing, with indoor-cured SF-EAM specimens as the control group.

**Figure 6:**
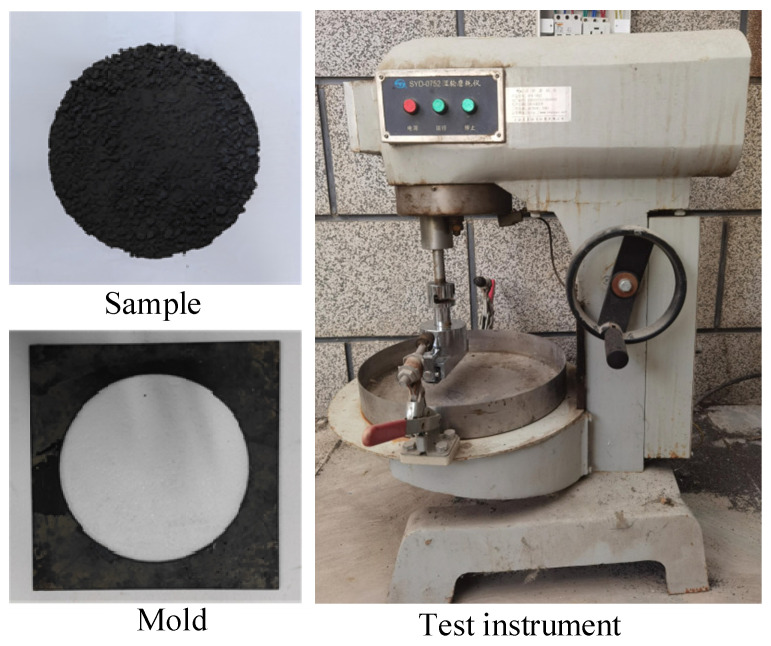
Wet wheel wear test set up.

**Figure 7:**
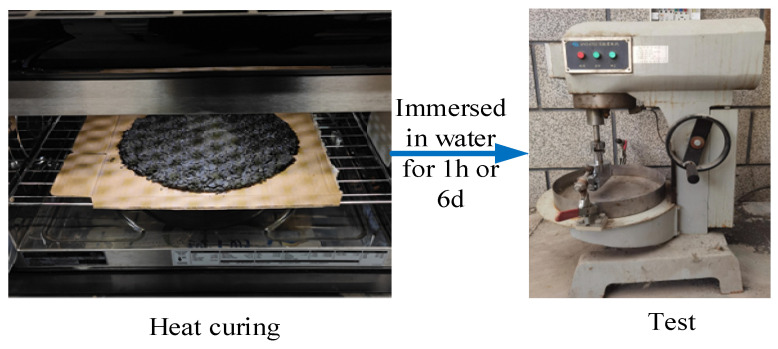
Wet wheel abrasion test after microwave heat curing.

The wet wheel wear test procedure is illustrated in Figure 6 and Figure 7. During the test, measure the combined mass of the oil felt and the specimen ($m_{a}$) before testing. Place the prepared specimen and oil felt in a 25 °C water bath for either 1 h or 6 days, and then install the specimen and operate the instrument. After the test, measure the total mass of the dried oil felt and specimen ($m_{b}$). The wear value is calculated using Equation (8):

(8) $$WTAT=(m_{a}-m_{b})/A$$

where WTAT is the wear value of the slurry mixture, g/m^2^; $m_{a}$ is the mass of the specimen before wear, g; $m_{b}$ is the mass of the specimen after wear, g; and A is the wear area of the abrasion head rubber tube, m^2^.

#### 2.2.5. Rutting Deformation Test

This section evaluates the anti-rutting performance of SF-EAM through wheel track deformation tests. Rutting is a permanent deformation that occurs under the combined effects of repeated driving loads and high temperatures, manifested as longitudinal strip-shaped grooves along the driving wheel tracks. Asphalt mixture has viscoelastic properties, and when subjected to repeated vehicle loads under high-temperature conditions, ruts may form due to the insufficient high-temperature stability of asphalt. As mentioned in the previous research, the microwave heat curing method can enhance the early strength of SF-EAM, and therefore, microwave heating will have a certain impact on the anti-rutting performance of SF-EAM. Two control experiments were set up:

1. The first group consisted of EAM and SF-EAM specimens after indoor curing, with EAM specimens as the control group.
2. The second group underwent experiments on SF-EAM after indoor curing and microwave heat curing, with indoor-cured SF-EAM specimens as the control group.

**Figure 8:**
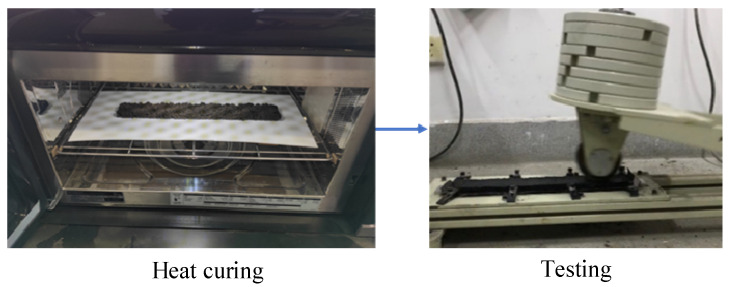
Wheel track deformation test after microwave heating and curing.

Complete the wheel track deformation test according to the standard procedures, as shown in Figure 8. After the experiment is completed, calculate the width deformation rate using Equation (9):

(9) $$PLD=(L_{b}-L_{a})\times 100/L_{a}$$

where PLD is the deformation rate per unit width of the specimen, %; $L_{a}$ is the initial width of the specimen before the test, mm; and $L_{b}$ is the width of the specimen after rolling, mm.

#### 2.2.6. Road Friction Coefficient Test

This section evaluates the anti-skid performance of SF-EAM through road friction coefficient testing. Asphalt pavement should have sufficient anti-skid performance to ensure that vehicles can travel safely at high speeds under the most unfavorable conditions, and its anti-skid performance should not quickly decrease under external factors. The surface structure of the road itself is the main provider of anti-skid performance, and the addition of SF can cause slight differences in the surface structure of SF-EAM. Therefore, it is necessary to study the anti-skid performance of SF-EAM. Two control experiments were set up:

1. The first group consisted of EAM and SF-EAM specimens after indoor curing, with EAM specimens as the control group.
2. The second group underwent experiments on SF-EAM after indoor curing and microwave heat curing, with indoor-cured SF-EAM specimens as the control group.

**Figure 9:**
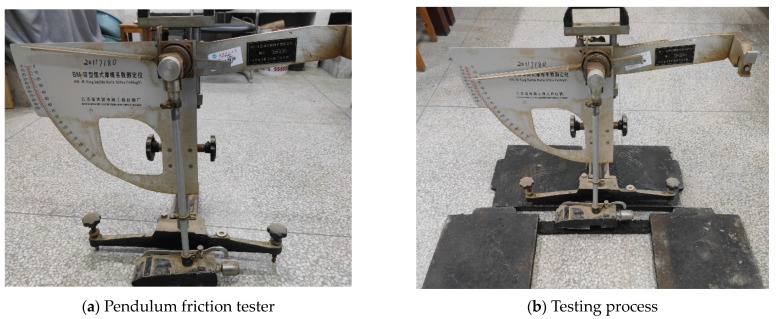
Road friction coefficient test.

Remove the cured specimen, fix it on both sides with thick steel plates, and clean the surface with a brush. Place the pendulum at the measuring point and set it to zero. Fix the pendulum on the right cantilever. Press the switch after spraying water on the test piece to make it scratch on the test piece. Repeat the operation to record data and note the surface temperature of the measuring point. The experimental process is shown in Figure 9. After the experiment is completed, correct the swing value for temperature according to Equation (10). The temperature correction values are shown in Table 8.

(10) $$BPN_{20}=BPN_{T}+\Delta BPN$$

where $BPN_{20}$ is the swing value converted to the standard temperature of 20 °C; $BPN_{T}$ is the swing value measured at the pavement temperature; and ΔBPN is the temperature correction value, obtained from Table 8.

## 3. Simulation Study on Microwave Absorption Performance of Emulsified Asphalt Mixture

### 3.1. Microwave Absorption Performance of Emulsified Asphalt Mixture

When using microwave heating to heat materials, it is essential for the material to contain a medium that can absorb microwaves. To quickly heat the emulsified asphalt mixture under microwave radiation, the mixture must include a microwave-absorbing medium. According to Equation (3), under the same external conditions, the microwave absorption performance of the emulsified asphalt mixture is related to its relative dielectric constant (*ε_r_*) and loss tangent value (tan*δ*). Emulsified asphalt mixture, as a composite medium, typically contains more than 80% mineral aggregate, 5–10% water, 4–8% petroleum asphalt, less than 10% mineral powder, and other additives. The dielectric constant and loss tangent values of common emulsified asphalt mixture materials are shown in Table 9 [30].

According to Table 9, the electromagnetic parameters of each component in the emulsified asphalt mixture are relatively low. Among them, the dielectric constant and loss tangent of pure asphalt are relatively small, indicating that it does not significantly absorb microwaves. The microwave absorption performance of aggregates is also similar to that of asphalt and is generally related to the metal mineral composition they contain, making the absorption performance unstable. The electromagnetic parameters of water are relatively high, providing good microwave absorption performance. However, the proportion of water in the emulsified asphalt mixture is small, resulting in an overall insignificant temperature rise during microwave heating.

Therefore, adding a high-performance microwave-absorbing material to the mixture, which can effectively absorb and attenuate incident electromagnetic waves, converting electromagnetic energy into thermal energy, can better leverage the advantages of microwave heating. According to the research of Chen et al. [9], SiC is an excellent electrical loss-type microwave-absorbing material with good impedance matching characteristics [31]. Fe_3_O_4_ is an excellent magnetic loss absorbing material with stronger microwave absorption performance than iron powder [32]. The electromagnetic parameters of SiC [33] and Fe_3_O_4_ [34] are shown in Table 10.

According to Table 10, the imaginary part of the dielectric constant ($\varepsilon^{\prime \prime }$) of SiC is very large, while the imaginary part of the magnetic permeability ($\mu^{\prime \prime }$) is almost zero. From Equation (4), it can be seen that the larger the imaginary part, the higher the electromagnetic loss. Therefore, SiC can effectively utilize the electrical energy in electromagnetic waves, whereas its ability to absorb and utilize magnetic energy is relatively weak. The properties of Fe_3_O_4_ are exactly the opposite of SiC. Both SiC and Fe_3_O_4_ can serve as excellent microwave-absorbing materials to enhance the microwave absorption performance of emulsified asphalt mixtures. However, since microwaves contain both electrical and magnetic energy, the loss mechanism of a single material cannot fully utilize the electromagnetic energy in microwaves.

Liu et al. [10] found that the reflection loss of the SiC-Fe_3_O_4_ composite material, composed of SiC and Fe_3_O_4_ in a certain proportion, is lower than that of single SiC or Fe_3_O_4_. Lower reflection loss allows more microwaves to penetrate into the interior of the material. Therefore, this study considered utilizing the different microwave loss mechanisms of the two materials simultaneously by introducing the SiC-Fe_3_O_4_ composite material and incorporating it into the emulsified asphalt mixture to improve the microwave absorption performance of the mixture.

### 3.2. Simulation of Microwave Heating Temperature Field for Emulsified Asphalt Mixture

#### 3.2.1. Constitutive Relationship between Microwave Field and Temperature Field

The electromagnetic field theory for the microwave heating of emulsified asphalt mixture involves solving Maxwell’s equations, which can be expressed in partial differential form, as shown in Equations (11) and (12).

Maxwell’s equations in differential form are as follows:

(11) ∇⋅D=ρ

(12) ∇⋅B=0

(13) $$\nabla \times E=-\frac{\partial B}{\partial t}$$

(14) $$\nabla \times H=J+\frac{\partial D}{\partial t}$$

where D is the electric displacement vector, C/m^2^; ρ is the charge density, C/m^3^; B is the magnetic flux density vector, T; E is the electric field vector, N/C; H is the magnetic field vector, A/m; and J is the current density, A/m^2^.

In order to better apply Maxwell’s equations, it is necessary to obtain constitutive relationships that describe the macroscopic behavior of matter under field influence. In assuming that emulsified asphalt mixture is an isotropic linear material, its constitutive equation can be formulated as shown in Equations (15)–(17):

(15) J=σE

(16) D=εE

(17) B=μH

where σ is the electrical conductivity of the material, S/m; ε is the permittivity of the material, F/m; and μ is the permeability of the material, H/m.

The formula for a Fourier transform is shown in Equation (15). According to the Fourier transform, the time domain in Maxwell’s equations is transformed into the frequency domain, as shown in Equation (18):

(18) $$f(\omega )=\int x\left(t\right)e^{-j\omega t}dt$$

(19) $$\frac{\partial }{\partial t}∼j\omega$$

According to Equations (18) and (19), the Helmholtz equation in the frequency domain can be derived from the Maxwell equations. The Helmholtz equation is an important control equation for electromagnetic waves when using COMSOL to solve microwave heating problems, as shown in Equation (20), where the expression for $k_{0}$ is shown in Equation (21):

(20) $$\nabla \times \mu_{r}^{-1}(\nabla \times E)-k_{0}^{2}(\varepsilon_{r}-j\frac{\sigma }{\omega \varepsilon_{0}})E=0$$

(21) $$k_{0}^{2}=\omega^{2}\mu_{0}\varepsilon_{0}$$

where $\mu_{0}$ is vacuum permeability, H/m; $\varepsilon_{0}$ is the vacuum dielectric constant, F/m; $\mu_{r}$ is the relative magnetic permeability; and $\varepsilon_{r}$ is the relative dielectric constant.

Microwave heating emulsified asphalt mixture generally transfers heat through thermal conduction. According to the theory of thermal conduction, the temperature distribution of emulsified asphalt mixture at various spatial points at any time is the temperature field distribution of the object.

The heat conduction problem of microwave heating of emulsified asphalt mixture can be expressed by the heat transfer equation, expressed in partial differential form as Equation (22). The heat transfer equation is an important control equation for solid heat transfer when using COMSOL to solve microwave heating problems.

(22) $$\rho C_{P}\frac{\partial T}{\partial t}+\rho C_{P}u\cdot \nabla T+\nabla \cdot (-k\nabla T)=Q+Q_{ted}$$

where ρ is the density of heated substance, kg/m^3^; $C_{P}$ is the constant pressure heat capacity, J/(kg·K); T is temperature, °C; k is thermal conductivity, W/(m·K); and Q is the heat source, J/(m^2^·s).

#### 3.2.2. Simulation Model of Microwave Heating Emulsified Asphalt Mixture

The coupling of multiple physical fields is generally divided into strong coupling and weak coupling. Weak coupling refers to the relatively weak mutual influence between physical fields, while strong coupling refers to the very intense mutual influence between physical fields [35]. The microwave heating problem of emulsified asphalt mixture specimens is a strong coupling problem between the electromagnetic wave field and the temperature field. The calculation process of the strong coupling problem model is very complex, and in the modeling process, it is necessary to simplify the complex problem to save computational costs. Therefore, this section makes the following assumptions about electromagnetic fields, temperature fields, materials, and geometric models:

1. During microwave heating, the thermal parameters such as heat capacity and electromagnetic parameters, such as the dielectric constant of emulsified asphalt mixture specimens, remain constant.
2. The influence of the internal electromagnetic field of emulsified asphalt mixture specimens is ignored.
3. After the microwave is absorbed by the emulsified asphalt mixture specimen, the microwave energy is completely converted into thermal energy.
4. The material inside the emulsified asphalt mixture specimen is homogeneous and isotropic.
5. The interior of the microwave oven is homogeneous and smooth, and does not absorb microwaves. Other materials inside the microwave oven do not absorb microwaves except for the specimen.

**Figure 10:**
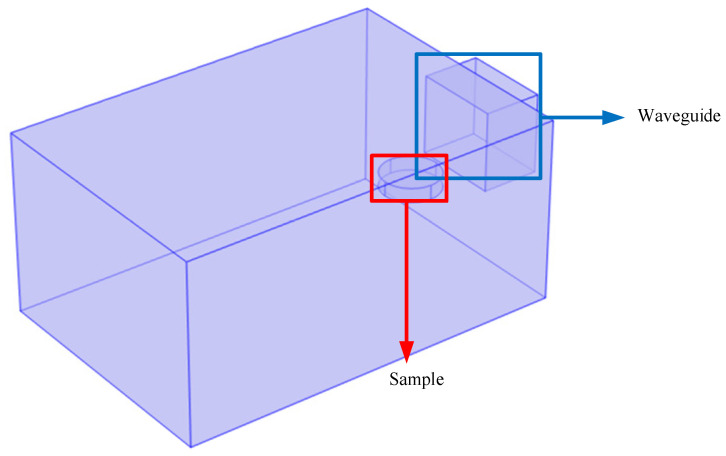
Geometric model of microwave oven and specimen.

In the subsequent microwave heating test, the emulsified asphalt mixture specimen was placed on a wooden thin plate. Due to the small volume of the wooden board and the low microwave dissipation of the wooden material, this part of the structure was ignored in the modeling process. The microwave oven cavity and waveguide are both rectangular prisms, with the dimensions of the microwave oven cavity being 0.53 m in length, 0.36 m in width, and 0.25 m in height. Regardless of the cavity thickness, the dimensions of the waveguide are 0.08 m in length, 0.12 m in width, and 0.11 m in height. The emulsified asphalt mixture specimen is cylindrical with a radius of 0.04 m and a height of 0.02 m. The specimen was placed at the rectangular waveguide mouth of a microwave oven and remained in a fixed position during the microwave heating process. The geometric models of the microwave oven, waveguide, and specimen are shown in Figure 10.

The microwave cavity was filled with air. Considering that the imaginary part of the dielectric constant of air is very small, it was assumed in the simulation that its imaginary part was 0, and the power loss caused by it was ignored. The surface resistance of the stainless-steel cavity is relatively small, and the electromagnetic loss of the cavity can be ignored. Therefore, it was assumed that there was no heat source or heat conduction in the area other than the emulsified asphalt mixture specimen during the heating process. There were four types of emulsified asphalt mixture specimens in this simulation: EAM, S-EAM (emulsified asphalt mixture mixed with SiC), F-EAM (emulsified asphalt mixture mixed with Fe_3_O_4_), and SF-EAM. Their electromagnetic and thermal parameters are shown in Table 11 and Table 12, respectively.

Define the waveguide and microwave cavity as impedance boundary conditions. The port boundary condition is used to define the boundary of microwave power entering the model. A heat flux boundary condition was set on the surface of the air in the microwave cavity in contact with the emulsified asphalt mixture specimen, and a universal value of 10 W/(m^2^·°C) was selected for the heat transfer coefficient [36]. The microwave power of the microwave oven was 200 W, and the microwave frequency was 2.45 GHz. Based on the existing microwave heating experience and actual laboratory conditions, the temperature of emulsified asphalt mixture specimens and air was set to 25 °C, and the microwave heating time was set to 120 s.

The mesh partitioning process is a crucial step in verifying the finite element model and establishing the reliability of the software, model, and computational results. Under electromagnetic waves at a frequency of 2.45 GHz, Liu et al. proposed a grid size calculation method [37], as shown in Equation (23):

(23) $$l=\frac{\lambda }{6\sqrt{\varepsilon^{\prime }}}$$

where l is the grid size, mm; λ is the wavelength of microwaves, mm; and $\varepsilon^{\prime }$ is the real part of the complex dielectric constant of a material.

**Figure 11:**
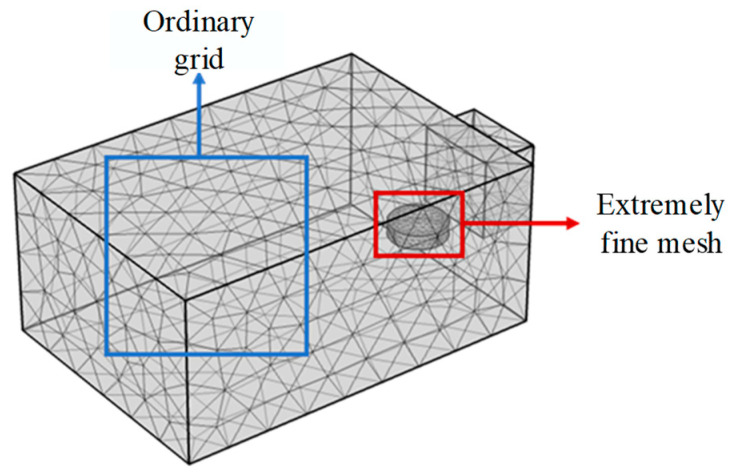
Grid division of microwave oven and specimen.

To accelerate the convergence of the simulation model and reduce unnecessary computation time, the main research object, emulsified asphalt mixture specimens, will be divided into extremely refined grids, while other areas will be divided into ordinary grids. The grid division of the specimens and other areas is shown in Figure 11. The number of grid cells in the emulsified asphalt mixture specimen after partitioning is 1571, and the number of grid cells in other parts is 9466. The minimum unit length ($l_{\min}$) of the emulsified asphalt mixture specimen grid partitioning is 1.22 × 10^−4^ m, and the maximum unit length ($l_{\max}$) is 0.0122 m, which conforms to Equation (23).

#### 3.2.3. Simulation Results of Microwave Heating Emulsified Asphalt Mixture

**Figure 12:**
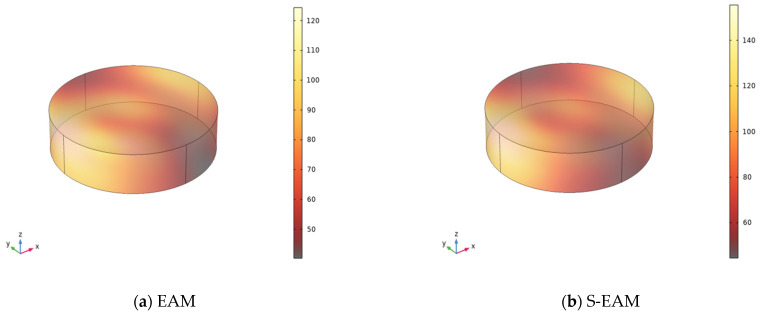

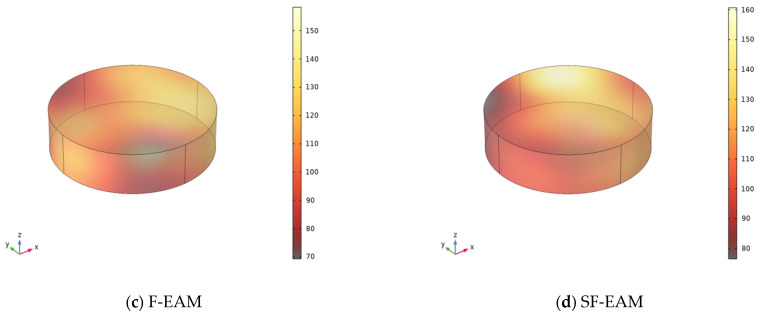
Temperature distribution of four materials after 120 s of microwave heating.

A simulation model for the microwave heating of emulsified asphalt mixture was established under the conditions of a microwave power of 200 W and a microwave frequency of 2.45 GHz. The temperature field distribution of four types of emulsified asphalt mixture specimens—EAM, S-EAM, F-EAM, and SF-EAM—after microwave heating was simulated. The influence of external materials on the temperature field change in emulsified asphalt mixture during microwave heating was studied, and the microwave absorption performances of different emulsified asphalt mixtures were compared. The simulation results are shown in Figure 12. The initial temperature of the specimen before heating was 25 °C, and the highest and lowest temperatures of the specimen after heating are shown in Table 13.

In Figure 12, the dark areas represent low-temperature regions, while the bright areas represent high-temperature regions. According to the data in Table 13 and the color comparison in Figure 12, it can be seen that after 120 s of microwave heating, the temperatures of all four emulsified asphalt mixtures increased. The temperature in most areas of EAM and S-EAM increased significantly, but the dark areas are also larger, with large temperature differences and uneven heating. The bright color areas of F-EAM and SF-EAM have a larger distribution range and more uniform heating. Among them, SF-EAM has the largest proportion of bright color areas and the greatest temperature rise.

**Figure 13:**
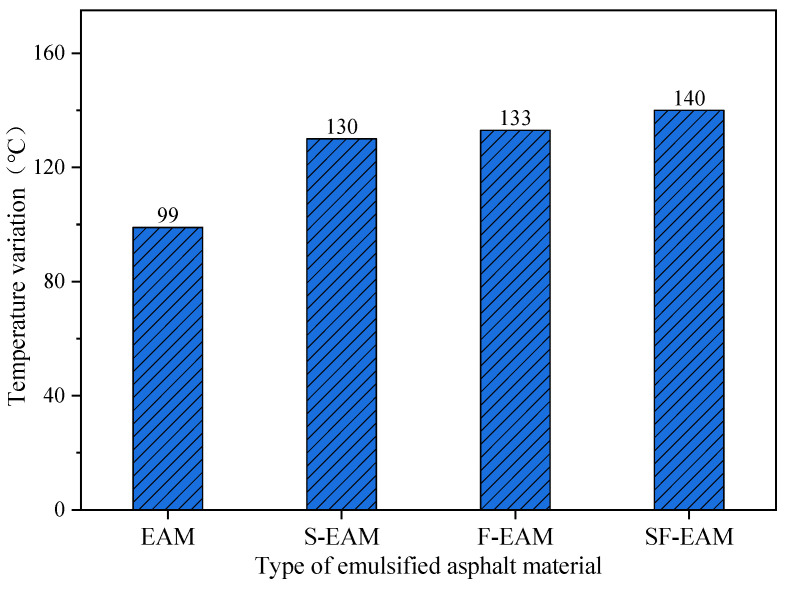
Temperature variation in different emulsified asphalt mixtures.

According to Table 13, a schematic of the temperature changes in different emulsified asphalt mixtures was drawn, as shown in Figure 13. The calculation method for temperature change is shown in Equation (24);

(24) $$\Delta T=T_{max}-T_{0}$$

where ΔT is the temperature variation, °C; $T_{max}$ is the maximum temperature, °C; and $T_{0}$ is the initial temperature, °C.

According to Table 13 and Figure 13, the temperatures of the four emulsified asphalt mixtures significantly increased after microwave heating. The temperature increases range from small to large as follows: EAM, S-EAM, F-EAM, and SF-EAM. The maximum temperature increases are 396%, 520%, 532%, and 560%, respectively. Compared to EAM, the temperature changes in S-EAM, F-EAM, and SF-EAM increased by 31%, 34%, and 41%, respectively. The maximum temperature differences in the four emulsified asphalt mixtures from small to large are EAM, SF-EAM, F-EAM, and S-EAM, but the maximum temperature differences are not significantly different.

When microwave heating emulsified asphalt mixtures of different compositions, the temperature increases. Among them, the temperature increase in emulsified asphalt mixtures mixed with SiC, Fe_3_O_4_, and SF is greater than that of ordinary emulsified asphalt mixtures, indicating that the addition of SiC and Fe_3_O_4_ can improve the microwave absorption performance of emulsified asphalt mixtures. The temperature increases in the emulsified asphalt mixture mixed with SF is the largest, and the microwave heating is more uniform, indicating that the improvement in microwave absorption performance by adding SF is more significant than adding a single material.

In summary, the microwave absorption performance of the material depends on the electric loss and magnetic loss characteristics of the material itself, and is related to the material’s relative dielectric constant ($\varepsilon_{r}$) and loss tangent value (tan*δ*). In the emulsified asphalt mixture, the electromagnetic parameters of each component material are relatively small, so its microwave absorption performance is poor. The real and imaginary parts of SiC permittivity are larger, and the real and imaginary parts of Fe_3_O_4_ permeability are larger. The SiC-Fe_3_O_4_ composite can improve the microwave absorption performance of emulsified asphalt mixture. Therefore, further research is needed on the curing performance of emulsified asphalt mixtures through microwave heating tests.

## 4. Results and Discussion of Microwave Curing Performance Test

### 4.1. Cohesion Test

The adhesion test results of EAM and SF-EAM are shown in Table 14.

**Figure 14:**
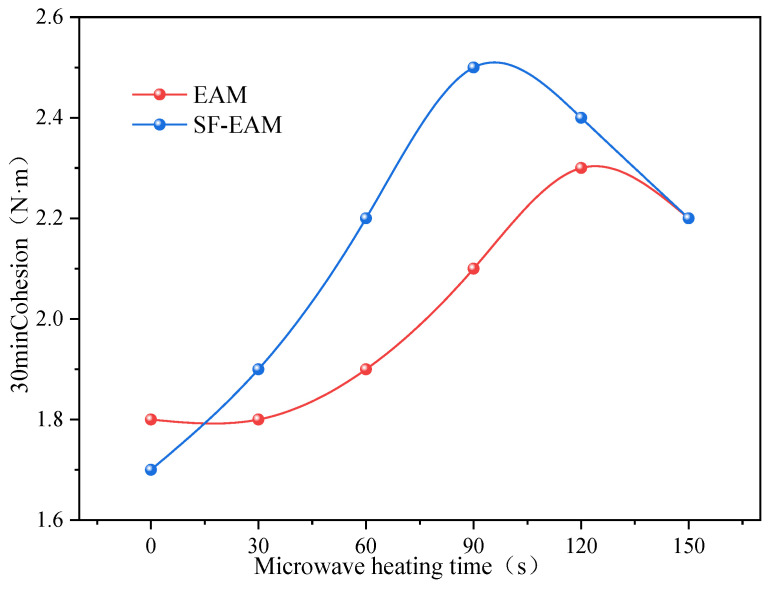
Thirty-minute cohesion of EAM and SF-EAM under different microwave heating times.

**Figure 15:**
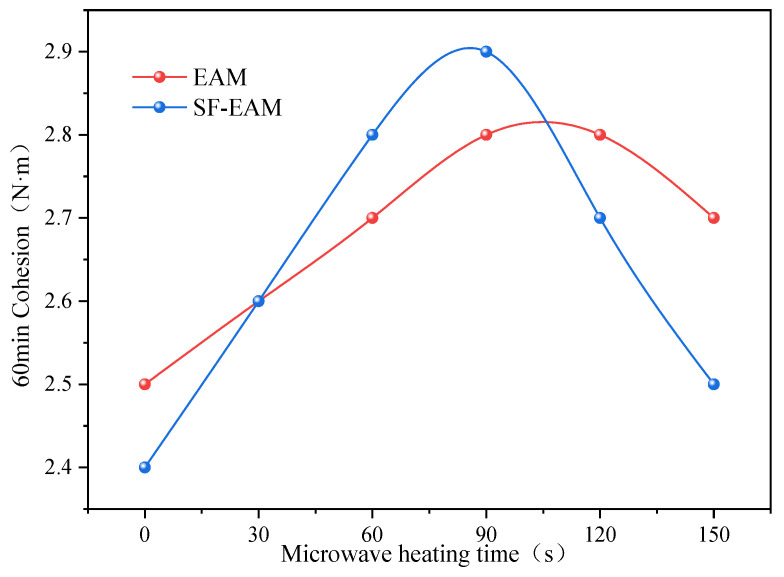
Sixty-minute cohesion of EAM and SF-EAM under different microwave heating times.

According to Table 14, the variation curves of the 30 min and 60 min cohesion of EAM and SF-EAM are plotted with different microwave heating times, as shown in Figure 14 and Figure 15.

From Figure 14 and Figure 15, it can be seen that as the microwave heating time increases; the 30 min cohesion of EAM and SF-EAM shows an initial increase followed by a decrease. The 30 min cohesion of EAM reaches its maximum value of 2.3 N·m after 120 s of microwave heating, while the 30 min cohesion of SF-EAM reaches its maximum value of 2.5 N·m after 90 s of microwave heating, indicating that the maximum 30 min cohesion of SF-EAM is greater and requires a shorter microwave heating time to reach its maximum value. As the microwave heating time increases, the 60 min cohesion of EAM and SF-EAM also shows an initial increase followed by a decrease. The 60 min cohesion of EAM reaches its maximum value of 2.8 N·m after 90 s of microwave heating, while the 60 min cohesion of SF-EAM reaches its maximum value of 2.9 N·m after 90 s of microwave heating, indicating that the 60 min cohesion of SF-EAM is even greater.

After microwave heating treatment, the internal temperatures of EAM and SF-EAM continuously increase with the increase in heating time. At high temperatures, the rate of water evaporation accelerates, and the specimens can be quickly cured and formed to a certain strength. Therefore, under the same total curing time, the longer the microwave heating time, the higher the strength of the specimens. However, the temperature of the emulsified asphalt mixture should not be too high. When the temperature exceeds 150 °C, the asphalt will gradually age due to high temperature. At this time, some of the light asphalt oil adhered to the aggregate will gradually evaporate, and the adhesion between asphalt and aggregate will deteriorate, resulting in a decrease in the strength of the emulsified asphalt mixture. Therefore, when the microwave heating time exceeds 120 s, both the 30 min and 60 min cohesion of SF-EAM will decrease.

Comparing EAM and SF-EAM emulsified asphalt mixtures, SF-EAM has a better ratio of mineral aggregate in EAM. Therefore, when cured directly without microwave heating, EAM has a greater cohesion force. However, SF-EAM contains microwave absorbing material SF, which has better microwave absorption performance than EAM. Therefore, after the same heating treatment time, SF-EAM absorbs more heat, increases temperature faster, evaporates moisture faster, heats and solidifies faster, and increases adhesion faster. Therefore, the time required for SF-EAM to reach its maximum cohesion is shorter. Correspondingly, the rate at which the cohesion of SF-EAM decreases is also faster than that of EAM.

**Figure 16:**
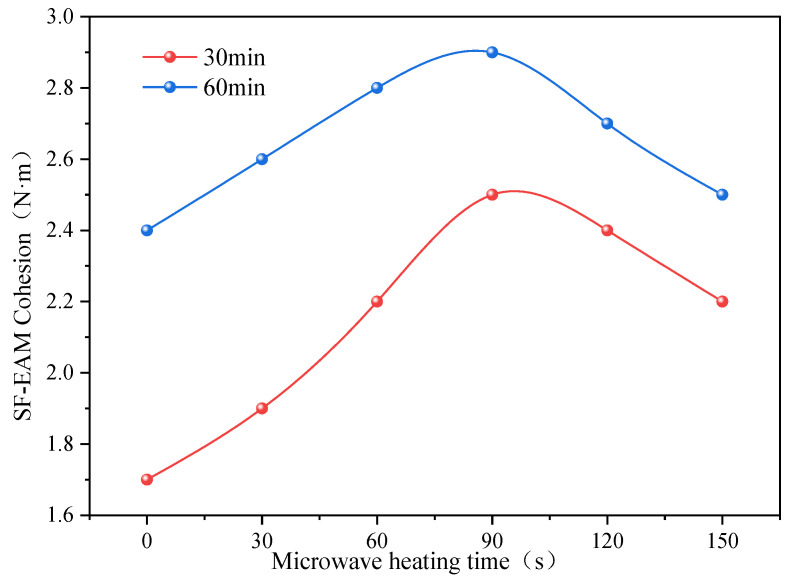
Cohesion force of SF-EAM under different microwave heating times.

According to Table 14, the comparison curves of the 30 min and 60 min cohesion of SF-EAM under different microwave heating times are drawn, as shown in Figure 16.

As shown in Figure 16, with the continuous increase in microwave heating time, the difference between the 30 min and 60 min cohesion of SF-EAM becomes smaller and smaller. Therefore, it can be concluded that the 30 min cohesion increase rate of SF-EAM after microwave heating is faster than the 60 min cohesion increase rate.

In comparing the 30 min and 60 min cohesion of SF-EAM, microwave heating has a greater impact on the 30 min cohesion of SF-EAM, resulting in a faster increase in the 30 min cohesion of SF-EAM. Microwave heating enhances the strength of emulsified asphalt mixture by accelerating the evaporation of water. When the total curing time increases from 30 min to 60 min, the water evaporation effect generated by microwave heating will be reduced. As the curing time gradually increases, the strength of SF-EAM cured by microwave heating will gradually approach that of SF-EAM cured by conventional curing, which also reflects that microwave heating has a greater impact on the early strength of SF-EAM.

Based on the adhesive strength test results of EAM and SF-EAM, microwave heating for a certain period of time can improve the early strength of both materials, but the improvement in SF-EAM is greater, indicating that the microwave heat curing performance of SF-EAM is better than that of EAM. When SF-EAM is microwave heated for 90 s, its 30 min and 60 min cohesion can reach their maximum values. At this time, the early strength of the emulsified asphalt mixture is relatively high. Therefore, the recommended microwave heating time for SF-EAM in this study is 90 s.

### 4.2. Wear Resistance Performance Test

The indoor curing process of EAM and SF-EAM was carried out in accordance with the standard specifications, and the microwave heat curing process referred to the previous text. After the experiment, calculate the 1 h wet wheel wear value of the emulsified asphalt mixture according to Equation (8), and the results are shown in Table 15.

According to Table 15, the 1 h wet wheel wear values of EAM and SF-EAM with different maintenance methods were all less than 540 g/m^2^, meeting the specification requirements. When both specimens are subjected to direct drying and heat curing, the wear value of SF-EAM is greater than that of EAM. When the curing method of SF-EAM specimens involves microwave heating, the wear value is lower than that of direct drying. The smaller the 1 h wear value, the smaller the wear loss of the emulsified asphalt mixture specimen, and the better the abrasion resistance of the specimen. Therefore, although the wear resistance of EAM under direct drying curing is better than that of SF-EAM, microwave heat curing can improve the wear resistance of SF-EAM. The anti-wear performance of SF-EAM after microwave heating is better than that of EAM.

Therefore, compared to EAM, SF-EAM contains 4% SF. The adhesion between SF and asphalt is not as good as that between mineral powder and asphalt. The overall compatibility of EAM is better than that of SF-EAM, resulting in less wear when subjected to external forces. However, after microwave heating and curing, the early strength of SF-EAM is improved, and the hard surface can better resist the wear of the wear head, thus enhancing its wear resistance.

### 4.3. Water Damage Resistance Performance Test

Water damage is usually caused by asphalt pavement damage in rainy areas in the south or during periods of freeze–thaw cycles in the north. Under the combined action of vehicles and loads, moisture gradually accumulates at the interface between asphalt and aggregate, causing the adhesion between asphalt and aggregate to gradually weaken over time. Like abrasion resistance, water damage resistance is also determined by the material properties and maintenance methods of the road surface.

This study evaluated the water damage resistance of SF-EAM through a 6-day wet wheel wear test. The experimental group setting method, specimen preparation and curing process, and microwave heat curing process refer to the previous text. After the experiment, calculate the 6-day wet wheel wear value of emulsified asphalt mixture according to Equation (8), and the results are shown in Table 16.

According to Table 16, the 6-day wet wheel wear values of EAM and SF-EAM with different maintenance methods were all less than 800 g/m^2^, meeting the regulatory requirements. When both specimens are subjected to direct drying and heat curing, the wear value of SF-EAM is greater than that of EAM. When the curing method of SF-EAM specimens involves microwave heating, the wear value is lower than that of direct drying. The smaller the 6-day wear value, the smaller the wear loss of the emulsified asphalt mixture specimen after immersion, and the better the water damage resistance of the specimen. Therefore, although the water damage resistance of EAM under direct drying curing is better than that of SF-EAM, microwave heat curing can enhance the water damage resistance of SF-EAM. The water damage resistance of SF-EAM after microwave heating is similar to that of EAM.

### 4.4. Anti-Rutting Performance

The experimental results are shown in Table 17.

According to Table 17, the width deformation rate of EAM and SF-EAM with different maintenance methods is less than 5%, meeting the requirements of the specifications. When both specimens are cured by direct drying, the width deformation rate of SF-EAM is greater than that of EAM. When the curing method of SF-EAM specimens involves microwave heating, the width deformation rate is smaller than that of direct drying. The smaller the width deformation rate, the smaller the rutting of the emulsified asphalt mixture under the load wheel gauge, and the better the anti-rutting performance of the specimen. Therefore, although the anti-rutting performance of EAM under direct drying curing is better than that of SF-EAM, microwave heat curing can improve the anti-rutting performance of SF-EAM. The anti-rutting performance of SF-EAM after microwave heating is slightly lower than that of EAM.

The particle size ratio of SF-EAM to EAM is coarser because the particle size of SF is coarser than the particle size of the mineral powder it replaces. Although a coarser grading generally has better anti-rutting performance, the coarse particles of SF-EAM are limited to the part below 0.075 mm in size, and the content of these coarse particles is very small, which has little effect on improving the anti-rutting performance. On the contrary, the particle size distribution of mineral powder in EAM is more uniform, making EAM denser than SF-EAM. Therefore, when directly dried, EAM has better anti-rutting performance. However, after microwave heating, the surface free asphalt of SF-EAM decreases, so microwave heating can improve the anti-rutting performance of SF-EAM.

### 4.5. Anti-Slip Performance Test

Due to the temperature being 25 °C during testing, the correction value for the swing value was taken as +2. The statistical test results after correction are shown in Table 18.

According to Table 18, the conversion values ($BPN_{20}$) of EAM and SF-EAM for different maintenance methods are all greater than 45, meeting the regulatory requirements. The difference in swing values between EAM and SF-EAM is not significant, and there is no significant difference in swing values between SF-EAM after microwave heating and after direct drying. Microwave heating has little effect on the anti-skid performance of SF-EAM.

The coarse and fine aggregate components of EAM and SF-EAM are identical, with differences only in the particles with a size of 0.075 mm or less. This fraction of the mineral material has little effect on the friction coefficient, resulting in similar swing values for EAM and SF-EAM. Although microwave heating can reduce the surface moisture content of SF-EAM, the swing value test involves spraying water on the surface of the specimen, and the surface moisture reduction from microwave heating can be largely ignored. Therefore, microwave heating has little effect on the swing value test results of SF-EAM.

## 5. Conclusions

This study investigated the microwave absorption simulation and curing performance of SiC-Fe_3_O_4_ emulsified asphalt mixture (SF-EAM). The main research conclusions are as follows:

- The SiC-Fe_3_O_4_ composite material (SF) formed by mixing SiC and Fe_3_O_4_ can improve the material’s relative dielectric constant (*ε_r_*) and loss tangent value (tan*δ*), thereby enhancing the microwave absorption performance of emulsified asphalt mixtures.
- The numerical simulation results from COMSOL show that compared to EAM, the temperature changes in S-EAM, F-EAM, and SF-EAM increased by 31%, 34%, and 41%, respectively. Therefore, the microwave absorption performance improvement effect of the SiC-Fe_3_O_4_ composite material is more significant than that of a single material.
- As the microwave heating time increases, the adhesion of SF-EAM first increases and then decreases. When microwave heating is 90 s, the 30 min and 60 min cohesion force of SF-EAM reach the maximum values of 2.5 N·m and 2.8 N·m, respectively. Therefore, the recommended microwave heating time for SF-EAM in this study is 90 s.
- After microwave heating and curing, the 1 h wet wheel wear value is 399.7 g/m^2^, the 6-day wet wheel wear value is 643.3 g/m^2^, the wheel track deformation rate is 4.21%, and the swing value of SF-EAM is 68°, which all meet the specifications. Therefore, microwave heating can not only improve the early strength of SF-EAM but also ensure that its road performance, such as wear resistance, water damage resistance, rutting resistance, and skid resistance, meets the requirements of the specifications.
- The particle size range of SiC and Fe_3_O_4_ used in this paper is 0.07~0.075 mm, and the influence of materials with other particle sizes on the microwave absorption performance of emulsified asphalt mixture was not considered. Therefore, further research is needed for materials with other particle sizes.

## Figures and Tables

**Table 1:** Technical performance of modified emulsified asphalt.

Test Item		Unit	Standard Specifications	Test Result	Test Method
Percentage of sieve residue		%	≤0.1	0.05	T0652
charge		/	Positive charge of cation	Positive charge of cation	T0653
Evaporative residue content		%	≥60	65	T0651
evaporative residue properties	penetration	0.1 mm	40~100	80	T0604
	softening point	°C	≥53	66	T0606
	ductility	cm	≥20	30	T0605
	solubility (Trifluoroethylene)	%	≥97.5	98	T0607
storage stability	1d	%	≤1	0.5	T0655
	5d	%	≤5	2.5	

**Table 2:** Technical performance of mineral materials.

Material	Test Item	Unit	Standard Specifications [15]	Test Result	Test Method
coarse aggregate	Stone crushing value	%	≤26	15	T0316
	Los Angeles attrition loss	%	≤28	20	T0317
	Stone polish value	BPN	≥42	50	T0321
	ruggedness	%	≤12	7	T0314
fine aggregate	Ruggedness	%	≤12	6	T0340
mineral aggregate	Equivalent of sand	%	≥65	78	T0334

**Table 3:** Technical performance of mineral powder.

Test Item	Unit	Standard Specifications [19]	Test Result	Test Method
Apparent relative density	t/m^3^	≥2.50	2.8	T0352
Water content	%	≤1	0.8	T0103
Particle size range < 0.6 mm	%	100	100	T0351
<0.15 mm	%	90~100	98	
<0.075 mm	%	75~100	92	
Coefficient of water affinity	/	<1	0.8	T0353
Index of plasticity	/	<4	3	T0354

**Table 4:** Technical properties of cement.

Test Item		Unit	Standard Specifications [21]	Test Result	Test Method
setting time	initial setting	min	≥45	132	T0505
	final setting	min	≤600	270	
compressive strength	3 d	MPa	≥17.0	20.4	T0553
	28 d	MPa	≥42.5	47.6	
flexural strength	3 d	MPa	≥4.0	5.3	T0558
	28 d	MPa	≥7.0	8.2	

**Table 5:** Technical performance of SiC.

Test Item	Unit	Standard Specifications [19]	Test Result	Test Method
Apparent relative density	t/m^3^	≥2.50	3.1	T0352
Water content	%	≤1	0.01	T0103
Particle size range < 0.6 mm	%	100	100	T0351
<0.15 mm	%	90~100	100	
<0.075 mm	%	75~100	95	
Coefficient of water affinity	/	<1	0.4	T0353
Index of plasticity	/	<4	3	T0354

**Table 6:** Technical performance of Fe_3_O_4_.

Test Item	Unit	Standard Specifications [19]	Test Result	Test Method
Apparent relative density	t/m^3^	≥2.50	4.7	T0352
Water content	%	≤1	0.05	T0103
Particle size range < 0.6 mm	%	100	100	T0351
<0.15 mm	%	90~100	100	
<0.075 mm	%	75~100	95	
Coefficient of water affinity	/	<1	0.6	T0353
Index of plasticity	/	<4	2.5	T0354

**Table 7:** Curing methods and curing time for each group of specimens.

Item	Thirty-Minute Adhesive Strength		Sixty-Minute Adhesive Strength	
	Microwave Heating Time (s)	Total Maintenance Time at Room Temperature (min)	Microwave Heating Time (s)	Total Maintenance Time at Room Temperature (min)
1	0	30	0	60
2	30	30	30	60
3	60	30	60	60
4	90	30	90	60
5	120	30	120	60
6	150	30	150	60

**Table 8:** Temperature correction values.

Temperature (°C)	0	5	10	15	20	25	30	35	40
Temperature correction value ΔBPN	−6	−4	−3	−1	0	+2	+3	+5	+7

**Table 9:** Dielectric constant and loss tangent of emulsified asphalt mixture materials.

Material		T/°C	f/MHz	$\varepsilon_{r}$	tanδ
Water		25	300	77.5	0.016
		25	3000	76.7	0.157
Pure asphalt		26	3000	2.5	0.001
Aggregate material diorite		20	2450	5.6~7	0.018~0.036
Asphalt mixture	Containing diorite	20	2450	5.8	0.034
	Containing limestone	20	2450	6.7	0.015
	Containing quartz	20	2450	4.0	0.006

**Table 10:** Electromagnetic parameters of several microwave absorbing materials.

Material	Electromagnetic Parameters (at 2.45 GHz Frequency)			
	$\varepsilon^{\prime }$	$\varepsilon^{\prime \prime }$	$\mu^{\prime }$	$\mu^{\prime \prime }$
SiC	42~45	7~10	1.2~1.25	0~0.1
Fe_3_O_4_	12~13	0.1~0.2	2.5~3	1.3~1.5

**Table 11:** Electromagnetic parameters of four emulsified asphalt mixtures.

Specimen	Conductivity (S/m)	$\varepsilon^{\prime }$	$\varepsilon^{\prime \prime }$	$\mu^{\prime }$	$\mu^{\prime \prime }$
EAM	8 × 10^−7^	6	0.25	1	0
S-EAM	8 × 10^−7^	6.34	0.33	1	0
F-EAM	8 × 10^−7^	6.05	0.28	1.21	0.04
SF-EAM	8 × 10^−7^	6.45	0.38	1.25	0.05

**Table 12:** Thermal parameters of four emulsified asphalt mixtures.

Specimen	Density ρ (kg/m^3^)	Thermal Conductivity k (W/(m·K))	Thermal Conductivity $C_{P}$ (J/(kg·K))
EAM	2500	1.5	800
S-EAM	2500	1.5	800
F-EAM	2500	1.5	800
SF-EAM	2500	1.5	800

**Table 13:** Temperature of four materials heating for 120 s.

Type	EAM	S-EAM	F-EAM	SF-EAM
Minimum temperature (°C)	40	50	69	77
Maximum temperature (°C)	124	155	158	165
Maximum temperature difference (°C)	84	105	89	88

**Table 14:** Cohesion force under different microwave heating times.

Specimen	Thirty-Minute Cohesion (N·m)		Sixty-Minute Cohesion (N·m)	
	EAM	SF-EAM	EAM	SF-EAM
1	1.8	1.7	2.5	2.4
2	1.8	1.9	2.6	2.6
3	1.9	2.2	2.7	2.8
4	2.1	2.5	2.8	2.9
5	2.3	2.4	2.8	2.7
6	2.2	2.2	2.7	2.5

**Table 15:** Test results of 1 h wet wheel wear test after immersion in water.

Specimen	Heat Curing Method	One-Hour Wear Value (g/m^2^)		
		First Value	Second Value	Average Value
EAM	Direct drying	402.2	399.5	400.9
SF-EAM	Direct drying	420.7	421.2	421.0
SF-EAM	Microwave heating	401.0	398.3	399.7

**Table 16:** Test results of wet wheel wear test after immersion in water for 6 days.

Specimen	Heat Curing Method	Six-Day Abrasion Value (g/m^2^)		
		First Value	Second Value	Average Value
EAM	Direct drying	644.5	628.8	636.7
SF-EAM	Direct drying	687.2	695.0	691.1
SF-EAM	Microwave heating	645.5	641.1	643.3

**Table 17:** Test results of wheel track deformation test.

Specimen	Heat Curing Method	Width Deformation Rate (%)		
		First Value	Second Value	Average Value
EAM	Direct drying	3.95	4.01	3.98
SF-EAM	Direct drying	4.22	4.41	4.32
SF-EAM	Microwave heating	4.15	4.26	4.21

**Table 18:** Test results of road friction coefficient test.

Specimen	Heat Curing Method	$\mathrm{Actual}\;\mathrm{Measured}\;\mathrm{Swing}\;\mathrm{Value}\;(BPN_{T}$)				$\mathrm{Convert}\;\mathrm{the}\;\mathrm{Swing}\;\mathrm{Value}\;(BPN_{20})$
		First Value	Second Value	Third Value	Average Value	
EAM	Direct drying	66.5	66.1	66.9	66.5	69
SF-EAM	Direct drying	65.2	67.1	66.4	66.2	68
SF-EAM	Microwave heating	66.0	66.1	65.8	66.0	68

## Data Availability

The original contributions presented in the study are included in the article, further inquiries can be directed to the corresponding author.
